# Differential cardiovascular and autonomic responses to structurally distinct intermittent hypoxia paradigms in rats

**DOI:** 10.1038/s41440-026-02588-7

**Published:** 2026-03-02

**Authors:** Sheng-Chieh She, Chi-Wei Lin, Chieh-Wen Chen, Cheng-Han Wu, Shiang-Suo Huang, Ching-Jung Lai, Terry B. J. Kuo, Ding-I Yang, Yi-Heng Hsie, Kuan-Liang Kuo, Cheryl C. H. Yang

**Affiliations:** 1 Institute of Brain Science, National Yang Ming Chiao Tung University, Taipei, Taiwan, ROC; 2 Sleep Research Center, National Yang Ming Chiao Tung University, Taipei, Taiwan, ROC; 3 Institute of Neuroscience, National Chengchi University, Taipei, Taiwan, ROC; 4 Research Center for Mind, Brain and Learning, National Chengchi University, Taipei, Taiwan, ROC; 5 Department of Information Management, National Taipei University of Nursing and Health Sciences, Taipei, Taiwan, ROC; 6 Department of Pharmacology and Institute of Medicine, Chung Shan Medical University, Taichung, Taiwan, ROC; 7 Department of Pharmacy, Chung Shan Medical University Hospital, Taichung, Taiwan, ROC; 8 Master Program in Biomedical Science, School of Medicine, Tzu Chi University, Hualien, Taiwan, ROC; 9 Department of Physiology, Tzu Chi University, Hualien, Taiwan, ROC; 10 Department of Education and Research, Taipei City Hospital, Taipei, Taiwan, ROC; 11 Tsaotun Psychiatric Center, Ministry of Health and Welfare, Nantou, Taiwan, ROC; 12 Department of Family Medicine, Taipei City Hospital Renai Branch, Taipei, Taiwan, ROC; 13 Institute of Biomedical Informatics, National Yang Ming Chiao Tung University, Taipei, Taiwan, ROC; 14 Brain Research Center, National Yang Ming Chiao Tung University, Taipei, Taiwan, ROC

**Keywords:** Intermittent hypoxia, Blood pressure, Autonomic regulation, Implemental hypertension

## Abstract

Intermittent hypoxia (IH), the key physiological stressor in obstructive sleep apnea, is commonly quantified by respiratory event frequency. However, clinical heterogeneity in hypertension among patients with comparable apnea-hypopnea index (AHI) suggests that episode timing, including the duration and frequency of desaturation-reoxygenation cycles, may exert distinct biological effects even under equal cumulative burden. To test this, male Wistar-Kyoto rats were exposed for 21 days (8 h/day) to IH with either 10-s hypoxia duration at 30 cycles/h (10s-30c) or 5-s hypoxia duration at 60 cycles/h (5s-60c), while room air served as a control. Cardiovascular regulation was evaluated by continuous measurement of mean arterial pressure, heart-rate variability, and baroreflex sensitivity, and broader systemic effects were assessed through sleep-wake architecture, EEG activity, spatial memory, and cortical/hippocampal protein markers. Both IH groups had elevated blood pressure and disrupted autonomic balance compared with controls. The 5s-60c group produced more sustained hypertension, blunted nocturnal dipping, greater baroreflex impairment, and enhanced beta power during sleep, indicating persistent sympathetic drive. By contrast, the 10s-30c group was associated with increased paradoxical sleep, impaired spatial memory, reduced NeuN expression, and stronger upregulation of IBA-1 and NF-κB. These findings demonstrate that equivalent cumulative hypoxic exposure with different temporal structures yields divergent cardiovascular and neurocognitive outcomes. High-frequency, short-duration episodes preferentially promoted cardiovascular dysregulation, whereas longer episodes were linked to neurocognitive vulnerability. Consideration of hypoxic episode duration may improve the mechanistic interpretation of cardiovascular heterogeneity associated with sleep-disordered breathing.

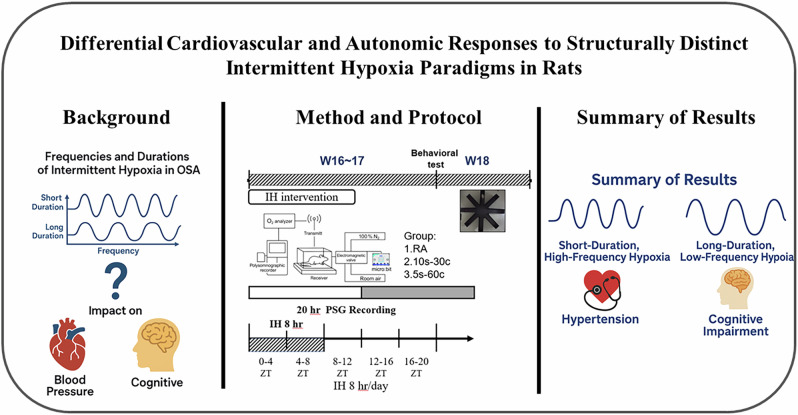

## Introduction

Obstructive sleep apnea (OSA) is highly prevalent among patients with cardiovascular disease and is strongly associated with hypertension, arrhythmia, myocardial infarction, and stroke [1]. A defining feature of OSA is intermittent hypoxia (IH), characterized by recurrent cycles of oxygen desaturation and reoxygenation during sleep [2]. These fluctuations activate the sympathetic nervous system, disrupt endothelial function, and drive sustained increases in blood pressure [3], thereby exacerbating adverse cardiovascular outcomes [4]. More than half of patients with OSA present with hypertension [5], underscoring the clinical relevance of IH as a cardiovascular stressor.

In clinical practice, OSA severity is commonly classified by the apnea-hypopnea index (AHI), which reflects the frequency of respiratory events but does not account for hypoxic episode duration [5]. This limitation is clinically significant, as cardiovascular complications such as resistant hypertension and heart failure do not develop uniformly among patients with comparable AHI values [1, 6, 7]. These heterogeneous outcomes suggest that the temporal structure of IH—specifically the duration and frequency of hypoxic episodes—may be an important determinant of cardiovascular risk.

Experimental studies demonstrate that IH provokes oxidative stress, inflammation, and autonomic dysregulation, processes that impair vascular and baroreflex function and contribute to sustained hypertension [8, 9]. At the same time, IH exerts systemic effects that extend to the central nervous system, where it has been associated with neuroinflammation, disrupted sleep architecture, and cognitive deficits [10–12]. These parallel pathways highlight the systemic nature of IH and may help explain the heterogeneous cardiovascular and neurocognitive outcomes observed in OSA patients. However, most prior models have relied on a single IH paradigm or limited their scope to either cardiovascular or neural outcomes, leaving unresolved whether distinct IH structures differentially shape these multi-level consequences.

The present study directly compared two IH paradigms with equivalent total hypoxic burden but different temporal structures: 30 cycles per hour with 10-s hypoxic episode duration (10s-30c) and 60 cycles per hour with 5-s episode duration (5s-60c). Here, “episode duration” refers to the duration of an individual hypoxic event at the target nadir, whereas “cycles per hour” denotes the frequency of hypoxic events within 1 h; together, these parameters define the temporal structure of each IH paradigm. Clinically, shorter but more frequent desaturation events resemble OSA subgroups characterized by high AHI but brief hypoxemia, whereas fewer but more prolonged events parallel patient phenotypes with elevated hypoxic burden despite lower event frequency. These clinical patterns conceptually correspond to the structural distinctions modeled in our 10s-30c and 5s-60c paradigms [13].

Using Wistar—Kyoto (WKY) rats, we systematically evaluated cardiovascular regulation (blood pressure, autonomic function, baroreflex sensitivity) complemented by assessments of sleep architecture, electroencephalography (EEG) activity, cognitive performance, and molecular markers of inflammation and oxidative stress. We hypothesized that the temporal structure of IH would differentially shape these systemic outcomes. Such insights may refine cardiovascular risk stratification in OSA by complementing AHI-based severity grading with physiologic indices such as hypoxic episode duration, thereby advancing mechanistic understanding of how temporal characteristics of intermittent hypoxia shape cardiovascular vulnerability.

## Materials and methods

### Animals and housing conditions

Thirteen-week-old male Wistar-Kyoto rats were housed under a 12:12-h light-dark cycle (lights on at 10:00 h) with free access to chow and water. For physiological monitoring, animals were implanted with wireless sensors and telemeters under general anesthesia, followed by ≥1 week of post-operative recovery. EEG, electromyography (EMG), and electrocardiography (ECG) were recorded using a custom-built wireless telemetry device, while arterial pressure was measured with an implantable telemeter. Sampling rates and filter settings followed established protocols. Animals received analgesia and antibiotics after surgery. Group sizes (*n* = 6–9) were based on pilot mean arterial pressure data during quiet sleep, providing >99% power (*α* = 0.05) to detect differences between IH group. All procedures were approved by the Institutional Animal Care and Use Committee (IACUC) of National Yang Ming Chiao Tung University (IACUC No. 1130408). Detailed housing conditions, surgical procedures, and statistical analyses are described in the Supplementary Methods [14].

### Surgical implantation procedures

EEG, EMG, ECG, and arterial pressure signals were processed for frequency-domain analysis, sleep stage classification, heart-rate variability (HRV), blood pressure variability, and baroreflex sensitivity according to established methods [14, 15]. Sleep stages were classified as awake (AW), quiet sleep (QS), or paradoxical sleep (PS) based on EEG and EMG features. Autonomic indices were derived from RR intervals, and the mean arterial pressure (MAP) and the low-frequency band of blood pressure variability (BLF) were calculated from arterial pressure waveforms [16–19] (see Supplementary Methods for details of data processing).

### Experimental protocol

A total of 37 male Wistar Kyoto rats were randomly assigned to one of three groups: 10s-30c (10-s hypoxic episodes at 30 cycles/hour for 8 h/day), 5s-60c (5-s episodes at 60 cycles/hour for 8 h/day), or room-air (RA) controls. The IH intervention lasted 3 weeks, with physiological recordings obtained during the second week and behavioral testing (open field and eight-arm maze) in the third week. At the end of the protocol, blood and brain tissues were collected for biochemical analyses. A detailed experimental timeline and representative raw traces of EEG, EMG, ECG, and arterial pressure for each group are provided in the Supplementary Methods (Figs. S1 and S2).

### IH model

The IH protocol was adapted from Fletcher et al. [20] To simulate the pattern observed in OSA. During the light phase, rats were exposed to cyclic hypoxia for 8 h/day over 21 days, with oxygen levels alternating between 21% and 4–6%. The hypoxic plateau was maintained for 5 or 10 s depending on group assignment. The cycle length was 1 min in the 5s–60c group and 2 min in the 10s-30c group, resulting in an identical total hypoxic duration across groups (see Supplementary Methods for chamber specifications and automated gas-control method) [20, 21].

To enhance translational clarity, the two IH paradigms were designed to dissociate event frequency from desaturation duration while maintaining an equivalent cumulative hypoxic dose. The 5s-60c paradigm produced shorter hypoxic episodes occurring at a higher frequency, conceptually analogous to OSA phenotypes characterized by a higher AHI but relatively brief desaturation events. In contrast, the 10s-30c paradigm generated fewer but longer desaturation periods, reflecting OSA patients who experience more prolonged hypoxemic episodes and thus a greater hypoxic burden per event despite a matched total hypoxic duration. This design allowed us to isolate the physiological impact of hypoxic event structure independent of total hypoxic exposure.

### Behavioral tests

#### Eight-arm maze test

Spatial learning was assessed using the eight-arm maze test. The primary behavioral outcomes were correct rate and error rate. Secondary measures included correct score (number of correct entries before the first error), total exploration time, number of arms visited, total correct entries, and number of errors (see Supplementary Methods for detailed definitions and procedures) [22].

### Collection of blood samples

Blood samples were collected from the carotid artery under isoflurane anesthesia prior to sacrifice for hematological and biochemical analyses. Detailed collection and processing procedures are described in the Supplementary Methods.

### Western blotting

Western blotting was used to assess cortical and hippocampal expression of nuclear factor kappa-light-chain-enhancer of activated B cells (NF-κB), glutathione peroxidase 4 (GPX4), glial fibrillary acidic protein (GFAP), cyclooxygenase-2 (COX-2), ionized calcium-binding adaptor molecule 1 (IBA-1), neuronal nuclei (NeuN), and brain-derived neurotrophic factor (BDNF), with glyceraldehyde-3-phosphate dehydrogenase (GAPDH) as the internal loading control. Detailed procedures and antibody specifications are provided in the Supplementary Methods.

### Statistical analyses

All data are presented as mean ± standard error of the mean (SEM). Outliers were identified using the boxplot method in IBM SPSS Statistics (IBM Corp., Armonk, NY), defined as values greater than 1.5 × the interquartile range (IQR) above the third quartile or below the first quartile, and were excluded prior to statistical testing. Group differences were analyzed using one-way analysis of variance (ANOVA). When the overall ANOVA was significant, Fisher’s least significant difference (LSD) test was applied for preplanned pairwise comparisons (10s-30c vs. RA, 5s-60c vs. RA, and 10s-30c vs. 5s-60c). A two-tailed *p*-value < 0.05 was considered statistically significant. Statistical analyses were performed using IBM SPSS Statistics (IBM Corp., Armonk, NY). Sample size was determined a priori based on MAP during the QS phase, which provided >99% post hoc power (*α* = 0.05, one-way ANOVA) to detect differences between IH paradigms. Accordingly, cardiovascular and autonomic parameters were designated as the primary endpoints. Sleep, cognitive, molecular, and hematological outcomes were analyzed as secondary or exploratory measures.

## Results

### Cardiovascular changes after 14 days of IH

#### Arterial pressure and vascular sympathetic nerve activity

**Fig. 1:**
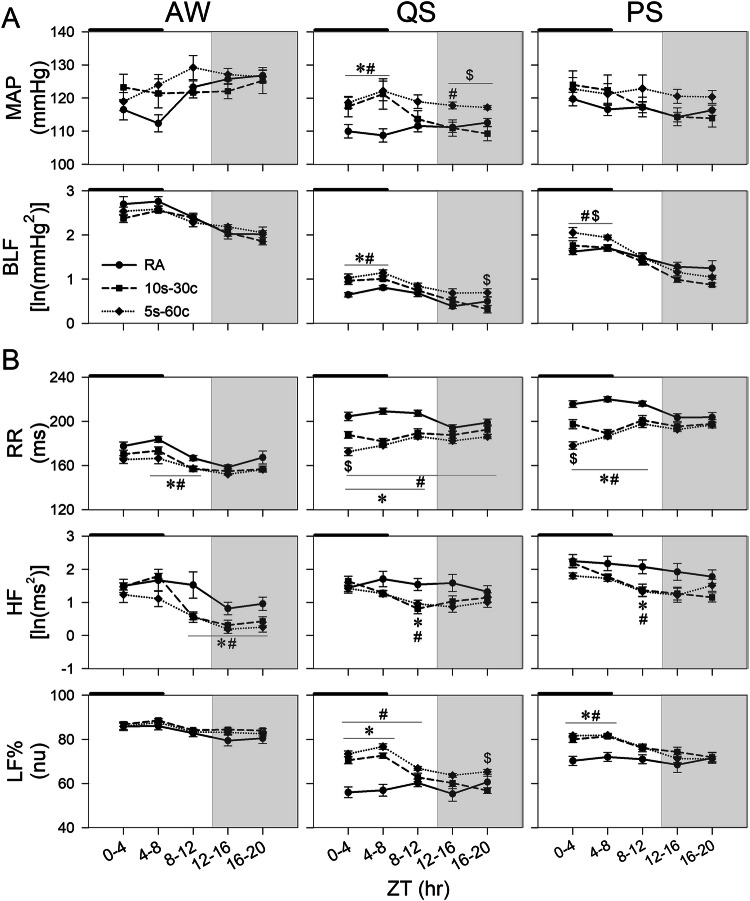
Time-course changes in cardiovascular and autonomic parameters. **A** 4-h interval changes in mean arterial blood pressure (MAP) and the low-frequency component of blood pressure variability (BLF). **B** 4-h interval changes in RR intervals (RR), high-frequency power of heart rate variability (HF), and normalized low-frequency power of heart rate variability (LF%) during awake (AW), quiet sleep (QS), and paradoxical sleep (PS) phases in the control, 10s-30c, and 5s-60c groups under and after intermittent hypoxia (IH) exposure. The black bar indicates the period of IH exposure, and the gray background indicates the dark phase. Data are presented as mean ± standard error of the mean. Line and symbol conventions: a solid line with circles represents the room-air control group (RA); a dashed line with squares represents the 10s-30c group; and a dotted line with diamonds represents the 5s-60c group. Groups: control (room air); 10s-30c (10-s episodes duration at 30 cycles/hour); 5s-60c (5-s episodes duration at 60 cycles/hour). Both IH protocols were administered for 8 h per day over 21 consecutive days. Sample sizes: **A** MAP and BLF: control (*n* = 9), 10s-30c (*n* = 8), and 5s-60c (*n* = 8); **B** RR, HF, and LF%: control (*n* = 13), 10s-30c (*n* = 11), and 5s-60c (*n* = 9). Statistical significance: *p < 0.05 for 10s-30c vs. control; ^#^*p* < 0.05 for 5s-60c vs. control; ^$^*p* < 0.05 for 5s-60c vs. 10s-30c (one-way analysis of variance [ANOVA] followed by Fisher’s least significant difference [LSD] post hoc test)

In QS, MAP was higher in both IH groups than in controls during IH exposure (ZT 0-8). Specifically, at ZT 0-4, MAP exceeded control values by 7.47 ± 3.69 mmHg in the 10s-30c group (*p* = 0.032) and by 8.65 ± 2.63 mmHg in the 5s-60c group (*p* = 0.019). At ZT 4-8, MAP remained higher in the 10s-30c and 5s-60c groups than in controls (Δ = 12.49 ± 5.04 mmHg, *p* = 0.006; Δ = 13.42 ± 3.59 mmHg, *p* = 0.027). During the post-IH period, MAP declined in the 10s-30c group to control levels, whereas it remained elevated in the 5s-60c group (ZT 0-4 vs. ZT 16-20: 118.63 ± 1.66 vs. 117.16 ± 0.63 mmHg; Δ = −1.47 ± 1.78), which exceeded the 10s-30c group at ZT 16-20 (Δ = 7.90 ± 2.28, *p* = 0.004). Similar trends were observed in PS (ZT 0-4 vs. ZT 16-20: 122.74 ± 3.44 vs. 120.34 ± 1.94 mmHg; Δ = −2.40 ± 3.95). increased in both IH groups during IH exposure and largely normalized post-exposure; however, at ZT 16-20, BLF remained higher in the 5s-60c group than in the 10s-30c group (Δ = 0.37 ± 0.12, *p* = 0.009), despite only modest within-group changes in the 5s-60c group from baseline to endpoint (1.03 ± 0.09 vs. 0.70 ± 0.09; Δ = −0.33 ± 0.12). In PS, BLF was higher in the 5s-60c group during IH exposure. At ZT 0-4 and ZT 4-8, BLF values in the 5s-60c group exceeded those in controls (ZT 0-4: Δ = 0.43 ± 0.13, *p* = 0.016; ZT 4-8: Δ = 0.24 ± 0.09, *p* = 0.043) and were also higher than those in the 10s-30c group (ZT 0-4: Δ = 3.01 ± 4.00, *p* = 0.041; ZT 4-8: Δ = 4.65 ± 3.62, *p* = 0.015). No post-exposure differences were observed at ZT 16-20. (Fig. 1A). Detailed mean ± SEM values at ZT 0-4 (during IH exposure) and ZT 16-20 (post-IH exposure), together with the corresponding differences, are summarized in Supplementary Table S1.

### Cardiac autonomic nervous system

In AW, both IH groups exhibited shorter RR intervals at ZT 4-8 and ZT 8-12 compared with RA. At ZT 4-8, RR intervals were reduced by −10.18 ± 4.30 ms in the 10s-30c group (*p* = 0.043) and by −17.22 ± 5.46 ms in the 5s-60c group versus RA (*p* = 0.002). At ZT 8-12, corresponding reductions were −9.75 ± 3.48 ms (*p* = 0.005) and −9.38 ± 2.86 ms (*p* = 0.008), respectively. During sleep, RR intervals were shorter in both IH groups during the exposure period across both sleep stages. In QS, RR intervals were reduced in the 10s-30c group (−16.82 ± 4.88 ms, *p* = 0.004) and the 5s-60c group (−32.00 ± 5.16 ms, *p* < 0.001). Similar reductions were observed during PS (10s-30c: −18.14 ± 5.18 ms, *p* < 0.001; 5s-60c: −37.54 ± 4.47 ms, *p* < 0.001). These effects persisted into the dark phase only in the 5s-60c group during QS (−12.82 ± 3.63 ms, *p* = 0.04) (Fig. 1B). Parasympathetic activity, assessed by the high-frequency power of HRV (HF), was unchanged during exposure but declined during the early post-exposure period, most prominently at ZT 8-12. At ZT 8-12, HF was reduced relative to RA by −0.73 ± 0.24 in the 10s-30c group (*p* = 0.003) and by −0.59 ± 0.21 in the 5s-60c group (*p* = 0.016). Sympathetic activity, assessed by the low-frequency normalized power of HRV (LF%), increased during exposure in both sleep phases. Post-exposure, LF% remained elevated only in the 5s-60c group, exceeding controls at ZT 8-12 (Δ = 6.58 ± 1.94) and the 10s-30c group at ZT 16-20 (Δ = 8.53 ± 1.71) (Fig. 1B). Summary statistics for the first and last analyzed time windows (ZT 0-4 and ZT 16-20) are provided in Supplementary Table S1.

### Baroreflex sensitivity

**Fig. 2:**
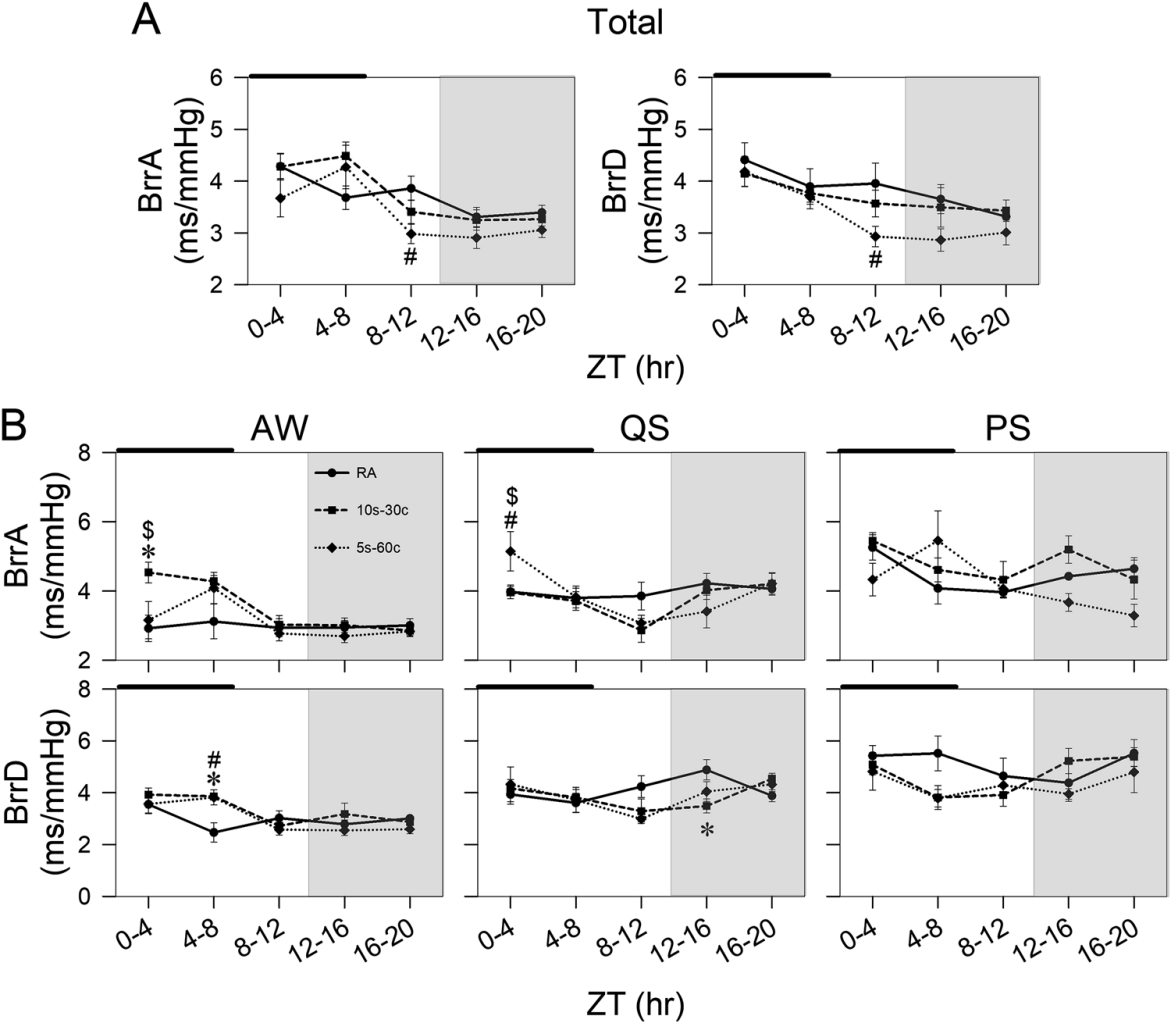
Time-course changes in baroreflex sensitivity. **A** 4-h interval changes in baroreflex sensitivity for ascending (BrrA) and descending (BrrD) across all vigilance states. **B** 4-h interval changes in BrrA and BrrD during awake (AW), quiet sleep (QS), and paradoxical sleep (PS) phases in the control, 10s-30c, and 5s-60c groups under and after intermittent hypoxia (IH) exposure. The black bar indicates the period of IH exposure. The gray background indicates the dark phase. BrrA reflects baroreflex sensitivity for sequences where mean arterial blood pressure (MAP) and RR intervals ascend concurrently, and BrrD reflects descending sequences. Line and symbol conventions: a solid line with circles represents the room-air control group (RA); a dashed line with squares represents the 10s-30c group; and a dotted line with diamonds represents the 5s-60c group. Groups: control (room air); 10s-30c (10-s episodes duration at 30 cycles/hour; and 5s-60c (5-s episodes duration at 60 cycles/hour). IH exposure was administered for 8 h per day over 21 consecutive days. Sample sizes: control (*n* = 8), 10s-30c (*n* = 8), and 5s-60c (*n* = 8). Statistical significance: *p < 0.05 for 10s-30c vs. control; ^#^*p* < 0.05 for 5s-60c vs. control; ^$^*p* < 0.05 for 5s-60c vs. 10s-30c (one-way analysis of variance [ANOVA] followed by Fisher’s least significant difference [LSD] post hoc test)

Across vigilance states, only the 5s-60c group exhibited reduced baroreflex sensitivity during both rising-pressure sequences (ascending index, BrrA; −0.88 ± 0.30, *p* = 0.008) and falling-pressure sequences (descending index, BrrD; −1.02 ± 0.44, *p* = 0.017) compared with controls at ZT 8-12, with no significant differences at other time points. During AW (ZT 0-4), BrrA was higher in the 10s-30c group than in both the control group (vs. RA: 1.62 ± 0.48, *p* = 0.0015) and the 5s-60c group (vs. 5s-60c: 1.38 ± 0.62, *p* = 0.028). In contrast, during QS, BrrA was higher in the 5s-60c group than in controls (vs. RA: 1.17 ± 0.60, *p* = 0.027) and the 10s-30c group (vs. 10s-30c: 1.19 ± 0.60, *p* = 0.025). No group differences in BrrA were observed post-IH (Fig. 2). BrrD increased in both IH groups during AW (ZT 4-8), with elevations of 1.39 ± 0.38 in the 10s-30c group (*p* = 0.002 vs. RA) and 1.35 ± 0.47 in the 5s-60c group (*p* = 0.002 vs. RA), but returned toward control levels after exposure. In QS, BrrD did not differ among groups during IH; however, during the post-IH period, BrrD was significantly lower in the 10s-30c group than in controls at ZT 12-16 (−1.39 ± 0.48, *p* = 0.01) (Fig. 2).

## Hematology analysis

Hematocrit (Δ = 5.28 ± 2.82, *p* = 0.048) and red cell distribution width (Δ = 1.53 ± 0.50, *p* = 0.019) were significantly higher in the 5s-60c group than in controls. Compared with the 10s-30c group, the 5s-60c group also exhibited higher hemoglobin (Δ = 2.69 ± 0.70, *p* = 0.005) and hematocrit levels (Δ = 8.51 ± 2.32, *p* = 0.002). White blood cell counts were lower in the 10s-30c group than in both controls (Δ = −1.64 ± 0.54, *p* = 0.018) and the 5s-60c group (Δ = −1.72 ± 0.62, *p* = 0.014). Platelet counts were likewise reduced in the 10s-30c group relative to controls (Δ = −110.32 ± 42.85, *p* = 0.023) and the 5s-60c group (Δ = −101.94 ± 49.33, *p* = 0.035) (Table 1). Other hematological and biochemical parameters did not differ significantly among groups (Supplementary Table S2).

**Table 1:** Comparison of Hematological Parameters among Different IH Interventions

			RA			10s-30c			5s-60c	
			mean	SE		mean	SE		mean	SE
RBC	10^6^/uL		7.05	0.53		6.67	0.38		7.45	0.30
HGB	g/dL		11.93	0.79		10.96	0.61		13.65^$^	0.36
HCT	%		34.05	2.06		30.82	1.29		39.32 ^* $^	1.93
WBC	10^3^/uL		4.13	0.51		2.49 ^*^	0.18		4.2 ^$^	0.60
PLT	10^3^/uL		489.50	28.43		379.18 ^*^	32.06		481.13^$^	37.49
RDW-CV	%		18.50	0.37		18.80	0.54		20.03 ^*^	0.33

Data are expressed as mean ± standard error (SE). Room air (RA; *n* = 11), control group exposed to normoxic room air; 10s-30c (*n* = 13), intermittent hypoxia (IH) with a 10-s hypoxia duration per 2-min cycle delivered at 30 cycles per hour; 5s-60c (*n* = 11), IH with a 5-s hypoxia duration per 1-min cycle delivered at 60 cycles per hour. Both IH protocols were applied for 8 h per day over 21 consecutive days. Abbreviations used in the table: RBC (red blood cell count), HGB (hemoglobin concentration), HCT (hematocrit), WBC (white blood cell count), PLT (platelet count), RDW-CV (red cell distribution width-coefficient of variation). Statistical significance: *p < 0.05 vs. RA; ^$^*p *< 0.05 vs. 10s-30c. One-way analysis of variance [ANOVA] followed by Fisher’s least significant difference [LSD] post hoc test was used for group comparisons

### Sleep changes after 14 days of IH

#### Sleep structure

**Fig. 3:**
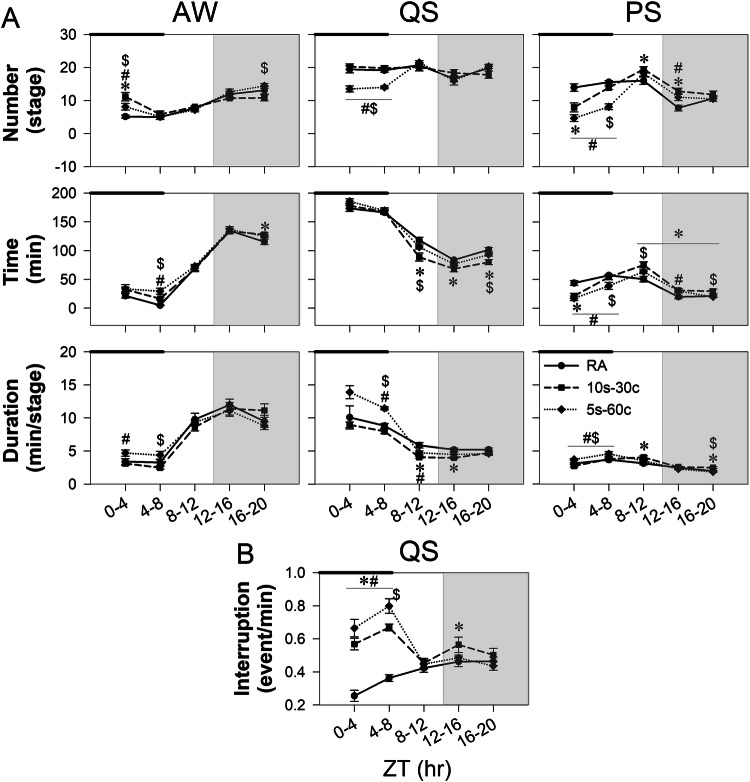
Time-course changes in sleep architecture. **A** 4-h interval changes in the number of sleep episodes, total sleep time, and average event duration during awake (AW), quiet sleep (QS), and paradoxical sleep (PS) phases. **B** 4-h interval changes in the number of sleep interruptions during the QS phase. The black bar indicates the period of intermittent hypoxia (IH) exposure, and the gray background indicates the dark phase. Data are presented as mean ± standard error of the mean. Line and symbol conventions: a solid line with circles represents the room-air control group (RA); a dashed line with squares represents the 10s-30c group; and a dotted line with diamonds represents the 5s-60c group. Groups: control (room air); 10s-30c (10-s episodes duration at 30 cycles/hour); 5s-60c (5-s episodes duration at 60 cycles/hour). Sample sizes: control (*n* = 13), 10s-30c (*n* = 12), and 5s-60c (*n* = 8). Statistical significance: *p < 0.05 for 10s-30c vs. control; ^#^*p* < 0.05 for 5s-60c vs. control; ^$^*p* < 0.05 for 5s-60c vs. 10s-30c (one-way analysis of variance [ANOVA] followed by Fisher’s least significant difference [LSD] post hoc test)

During the AW stage at ZT 0-4, both IH groups showed a higher number of AW events than controls (10s-30c: Δ = 6.01 ± 1.22, *p* < 0.001; 5s-60c: Δ = 2.97 ± 0.48, *p* = 0.047), with the increase being greater in the 10s-30c group than in the 5s-60c group (Δ = 3.04 ± 1.62, *p* = 0.045). In contrast, for total AW time, an increase relative to controls was observed only in the 5s-60c group (Δ = 1.29 ± 0.44, *p* = 0.038). At ZT 4-8, total AW time was higher in the 5s-60c group than in both controls (Δ = 13.57 ± 2.26, *p* = 0.006) and the 10s-30c group (Δ = 13.02 ± 5.74, *p* = 0.01); a corresponding increase was also observed in AW duration relative to the 10s-30c group (Δ = 1.88 ± 0.63, *p* = 0.005). Following IH exposure, group differences in AW time were limited. At ZT 16-20, total AW time was higher in the 10s-30c group than in controls (Δ = 12.74 ± 4.80, *p* = 0.014); however, the number of AW events in this group was lower than in the 5s-60c group (Δ = −3.83 ± 1.20, *p* = 0.017) (Fig. 3A).

During QS, the number of QS events was lower in the 5s-60c group than in both controls and the 10s-30c group at ZT 0-4 (vs. RA: Δ = −5.93 ± 1.05, *p* < 0.001; vs. 10s-30c: Δ = −6.75 ± 1.24, *p* < 0.001) and ZT 4-8 (vs. RA: Δ = −5.14 ± 0.56, *p* < 0.001; vs. 10s-30c: Δ = −5.80 ± 0.80, *p* < 0.001). For total QS time, a reduction relative to controls was observed only in the 10s-30c group after IH exposure at ZT 8-12 (Δ = −29.35 ± 5.63, *p* < 0.001), which persisted through ZT 16-20 (Δ = −21.60 ± 4.48, *p* < 0.001). Total QS time in the 10s-30c group was also lower than in the 5s-60c group at ZT 8-12 (Δ = −16.93 ± 7.91, *p* = 0.037) and ZT 16-20 (Δ = −11.73 ± 5.24, *p* < 0.001). With respect to QS event duration, values were higher in the 5s-60c group than in both controls and the 10s-30c group at ZT 4-8 (vs. RA: Δ = 2.65 ± 0.33, *p* < 0.001; vs. 10s-30c: Δ = 3.49 ± 0.43, *p* < 0.001). After IH exposure, QS duration was lower in the 10s-30c group than in controls at ZT 8-12 (Δ = −1.80 ± 0.58, *p* = 0.001) and ZT 12-16 (Δ = −1.24 ± 0.49, *p* = 0.002). In addition, at ZT 8-12, QS duration in the 5s-60c group was lower than in controls (Δ = −1.17 ± 0.41, *p* = 0.025). Regarding QS interruption, both IH groups showed higher values than controls at ZT 0-4 (10s-30c: Δ = 0.31 ± 0.19, *p* < 0.001; 5s-60c: Δ = 0.41 ± 0.03, *p* < 0.001) and ZT 4-8 (10s-30c: Δ = 0.31 ± 0.14, *p* < 0.001; 5s-60c: Δ = 0.44 ± 0.02, *p* < 0.001). At ZT 4-8, QS interruption was higher in the 5s-60c group than in the 10s-30c group (Δ =  0.13 ± 0.05, *p* = 0.004). After IH exposure, an increase in QS interruption was observed only in the 10s-30c group at ZT 12-16 relative to controls (Δ = 0.10 ± 0.22, *p* = 0.036) (Fig. 3A, B).

During PS, the number of PS events was lower in both IH groups than in controls at ZT 0-4 (10s-30c: Δ = −6.01 ± 1.63, *p* = 0.001; 5s-60c: Δ = −9.26 ± 1.13, *p* < 0.001), with no difference between the two IH groups. At ZT 4-8, this reduction persisted only in the 5s-60c group, which remained lower than both controls (Δ = −7.46 ± 0.50, *p* < 0.001) and the 10s-30c group (Δ = −5.79 ± 1.03, *p* < 0.001). After IH exposure, the number of PS events increased in the 10s-30c group at ZT 8-12 (Δ =  3.45 ± 1.42, *p* = 0.015) and ZT 12-16 (Δ = 5.05 ± 1.31, *p* = 0.001), whereas an increase in the 5s-60c group was observed only at ZT 12-16 (Δ = 3.25 ± 0.88, *p* = 0.023). Total PS time showed parallel changes during IH exposure, with reductions in both IH groups at ZT 0-4 (10s-30c: Δ = −22.45 ± 4.63, *p* < 0.001; 5s-60c: Δ = −26.47 ± 4.12, *p* < 0.001). At ZT 4-8, total PS time remained lower only in the 5s-60c group than in controls (Δ = −18.30 ± 1.95, *p* = 0.003) and the 10s-30c group (Δ =  −15.14 ± 7.55, *p* = 0.022). After IH exposure, total PS time increased in the 10s-30c group at ZT 8-12 (Δ = 24.78 ± 5.26, *p* < 0.001) relative to controls, and values in the 10s-30c group exceeded those in the 5s-60c group at ZT 16-20 (Δ = 8.52 ± 2.80, *p* = 0.023). In the 5s-60c group, total PS time was higher than controls at ZT 12-16 (Δ = 10.26 ± 3.08, *p* = 0.031) but was lower than that in the 10s-30c group at ZT 16-20 (Δ = −9.52 ± 3.95, *p* = 0.01). For PS event duration, values were higher in the 5s-60c group than in both controls (ZT 0-4: Δ = 0.60 ± 0.14, *p* = 0.016; ZT 4-8: Δ = 0.87 ± 0.10, *p* = 0.009) and the 10s-30c group (ZT 0-4: Δ = 0.96 ± 0.22, *p* < 0.001; ZT 4-8: Δ = 0.79 ± 0.41, *p* = 0.019). After IH exposure, PS duration increased in the 10s-30c group at ZT 8-12 (Δ = 0.90 ± 0.54, *p* = 0.004) and ZT 16-20 (Δ = 0.51 ± 0.38, *p* = 0.002); at ZT 16-20, PS duration in the 10s-30c group was higher than in the 5s-60c group (Δ = 0.64 ± 0.16, *p* = 0.01) (Fig. 3A).

#### EEG patterns

**Fig. 4:**
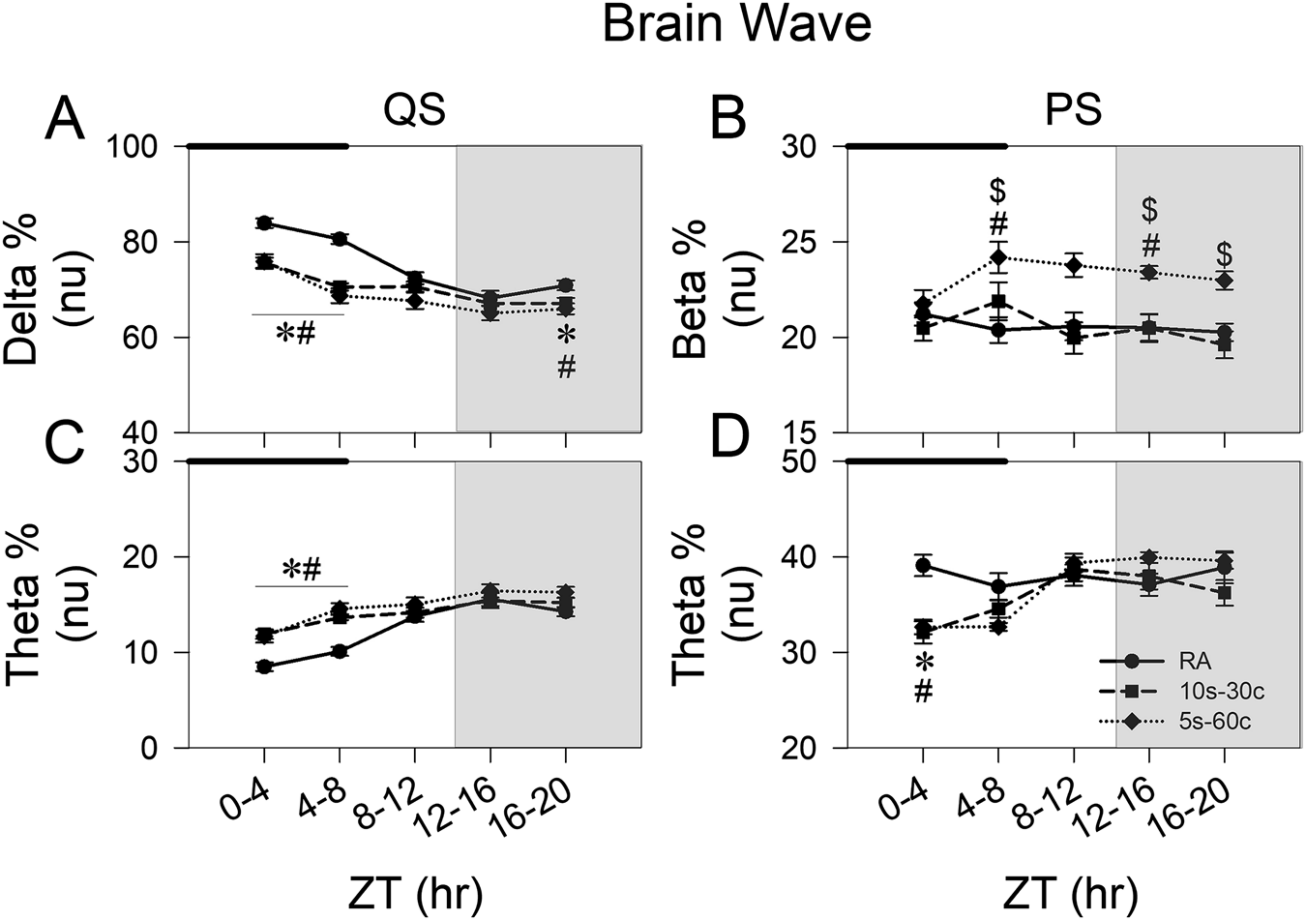
Electroencephalography (EEG) spectral power during sleep. Four-hour interval changes in delta%, beta%, and theta% during quiet sleep (QS) and paradoxical sleep (PS) phases in the control, 10s-30c, and 5s-60c groups under and after intermittent hypoxia (IH) exposure. **A** Delta% during QS; **B** beta% during PS; **C** theta% during QS; and **D** theta% during PS. The black bar indicates the period of IH exposure, and the gray background indicates the dark phase. Data are presented as mean ± standard error of the mean. Line and symbol conventions: a solid line with circles represents the room-air control group (RA); a dashed line with squares represents the 10s-30c group; and a dotted line with diamonds represents the 5s-60c group. Groups: control (room air); 10s-30c (10-s episodes duration at 30 cycles/hour); 5s-60c (5-s episodes duration at 60 cycles/hour). Sample sizes: control (*n* = 13), 10s-30c (*n* = 11), and 5s-60c (*n* = 9). Statistical significance: *p < 0.05 for 10s-30c vs. control; ^#^*p* < 0.05 for 5s-60c vs. control; ^$^*p* < 0.05 for 5s-60c vs. 10s-30c (one-way analysis of variance [ANOVA] followed by Fisher’s least significant difference [LSD] post hoc test)

During IH exposure, delta power during QS was reduced in both IH groups at ZT 0-4 (10s-30c: Δ = −8.31 ± 1.49, *p* < 0.001; 5s-60c: Δ = −8.03 ± 1.82, *p* < 0.001) and ZT 4-8 (10s-30c: Δ = −10.01 ± 1.29, *p* < 0.001; 5s-60c: Δ = −11.85 ± 1.86, *p* < 0.001). Theta power during PS was also reduced in both IH groups at ZT 0-4 (10s-30c: Δ = −7.01 ± 1.60, *p* < 0.001; 5s-60c: Δ = −6.44 ± 1.35, *p* < 0.001). These changes largely resolved after IH exposure, except at ZT 16-20, when QS delta power remained lower than controls in both IH groups (10s-30c: Δ = −3.75 ± 1.55, *p* = 0.021; 5s-60c: Δ =  −4.91 ± 1.55, *p* = 0.005) (Fig. 4A, D). In contrast, QS theta power was higher during IH exposure at ZT 0-4 (10s-30c: Δ = 3.41 ± 0.66, *p* < 0.001; 5s-60c: Δ = 3.20 ± 0.80, *p* < 0.001) and ZT 4-8 (10s-30c: Δ = 3.54 ± 0.53, *p* < 0.001; 5s-60c: Δ = 4.47 ± 0.74, *p* < 0.001) (Fig. 4C). Beta power during PS was selectively higher in the 5s-60c group, exceeding both controls and the 10s-30c group at ZT 4-8 (vs. RA: Δ = 3.81 ± 1.06, *p* = 0.01; vs. 10s-30c: Δ = 2.30 ± 1.29, *p* = 0.024). This increase persisted into the dark period at ZT 12-16 (vs. RA: Δ = 2.90 ± 0.76, *p* = 0.005; vs. 10s-30c: Δ = 2.91 ± 0.81, *p* = 0.001) and ZT 16-20 (vs. 10s-30c: Δ = 3.37 ± 0.85, *p* = 0.018) (Fig. 4B).

### Cognitive behavioral results after 14 days of IH

#### Eight-arm maze test

In the eight-arm maze, the 10s-30c group exhibited more total arm entries than controls (Δ = 3.33 ± 1.64, *p* = 0.027) and the 5s-60c group (Δ = 4.83 ± 1.73, *p* = 0.004). This group also showed a lower percentage of correct entries compared with controls (Δ = −12.47 ± 4.19, *p* = 0.02) and the 5s-60c group (Δ = −13.26 ± 6.18, *p* = 0.018). In addition, the number of wrong arm entries was higher in the 10s-30c group than in controls (Δ = 4.08 ± 1.74, *p* = 0.013) and the 5s-60c group (Δ = 5.75 ± 1.84, *p* = 0.002). The error percentage was also higher in the 10s-30c group compared with the 5s-60c group (Δ = 14.68 ± 5.95, *p* = 0.009) (Supplementary Fig. S3).

### Western blot

After 21 days of IH exposure, cortical GFAP and NF-κB expression levels were significantly increased in both IH groups compared with controls (GFAP: 10s-30c vs. RA, Δ = 0.56 ± 0.16, *p* < 0.001; 5s-60c vs. RA, Δ = 0.42 ± 0.08, *p* = 0.003; NF-κB: 10s-30c vs. RA, Δ = 0.49 ± 0.18, *p* = 0.022; 5s-60c vs. RA, Δ = 0.52 ± 0.21, *p* = 0.031), whereas COX-2 expression was reduced in both IH groups (10s-30c vs. RA, Δ = −0.51 ± 0.11, *p* < 0.001; 5s-60c vs. RA, Δ = −0.34 ± 0.10, *p* = 0.008). Neuronal markers exhibited divergent patterns between IH groups. In the 10s-30c group, cortical NeuN and proBDNF levels were reduced compared with both controls (NeuN: Δ = −0.47 ± 0.09, *p* = 0.004; proBDNF: Δ = −0.32 ± 0.09, *p* = 0.04) and the 5s-60c group (NeuN: Δ = −0.94 ± 0.14, *p* < 0.001; proBDNF: Δ = −0.78 ± 0.28, *p* = 0.015). In contrast, the 5s-60c group exhibited higher NeuN expression than controls (Δ = 0.47 ± 0.17, *p* = 0.003). In addition, cortical IBA-1 expression was lower in the 10s-30c group compared with the 5s-60c group (Δ = −0.28 ± 0.09, *p* = 0.004). In the hippocampus, GFAP, NF-κB, and GPX4 expression levels were increased in both IH groups relative to controls (GFAP: 10s-30c vs. RA, Δ = 0.33 ± 0.11, *p* = 0.008; 5s-60c vs. RA, Δ = 0.32 ± 0.11, *p* = 0.007; NF-κB: 10s-30c vs. RA, Δ = 0.29 ± 0.10, *p* = 0.026; 5s-60c vs. RA, Δ = 0.32 ± 0.13, *p* = 0.014; GPX4: 10s-30c vs. RA, Δ = 0.37 ± 0.09, *p* = 0.025; 5s-60c vs. RA, Δ = 0.46 ± 0.17, *p* = 0.006). In contrast, hippocampal NeuN and IBA-1 expression were reduced in both IH groups compared with controls (NeuN: 10s-30c vs. RA, Δ = −0.64 ± 0.08, *p* < 0.001; 5s-60c vs. RA, Δ = −0.17 ± 0.05, *p* = 0.021; IBA-1: 10s-30c vs. RA, Δ = −0.55 ± 0.10, *p* = 0.001; 5s-60c vs. RA, Δ = −0.70 ± 0.10, *p* < 0.001). Notably, the reduction in hippocampal NeuN was more pronounced in the 10s-30c group than in the 5s-60c group (Δ = −0.47 ± 0.07, *p* < 0.001) (Supplementary Fig. S4).

## Discussion

Unlike most preclinical IH models that vary total exposure or simply contrast IH with normoxia, this study fixed cumulative hypoxic time to isolate episode structure. This approach addresses a key gap in clinical metrics, as AHI reflects event frequency but not duration. Comparing the 10s-30c paradigms, characterized by longer but fewer hypoxic episodes, with the 5s-60c paradigms, defined by shorter and more frequent episodes, we found that delivery pattern critically shaped outcomes. The 5s-60c paradigm produced sustained sympathetic activation, higher MAP during QS, and impaired sleep continuity, whereas 10s-30c caused milder cardiovascular effects but greater neuronal vulnerability. These divergent phenotypes under equal hypoxic load show that temporal structure is a biologically meaningful factor that contributes to understanding heterogeneity in cardiovascular responses associated with OSA. Subsequent sections outline effects on blood pressure, autonomic regulation, sleep, and molecular responses.

### Sustained sympathetic activity and blood pressure elevation

Persistent autonomic imbalance was a common feature of both IH paradigms, but the magnitude and pattern differed by structure. During the dark phase, MAP elevation was greater in the 5s-60c group, accompanied by blunted nocturnal dipping during QS. This profile was consistent with higher LF% and shorter RR intervals, indicating sustained sympathetic drive. Given that attenuated nocturnal dipping is a recognized marker associated with cardiovascular risk, these findings suggest that high-frequency IH is associated with a vascular–autonomic phenotype characterized by persistent impairment of blood pressure regulation beyond hypoxia exposure [23–26].

### Baroreflex sensitivity as a marker of cardiovascular risk

Depressed baroreflex sensitivity is a strong predictor of arrhythmias and sudden death [27]. In the Autonomic Tone and Reflexes After Myocardial Infarction (ATRAMI) study, patients with myocardial infarction (MI) who had baroreflex sensitivity values below 3 ms·mmHg⁻¹ exhibited more than a fourfold increase in arrhythmic mortality risk, independent of ventricular function or HRV [28, 29]. Extending this, our data show baroreflex impairment distinguished IH paradigms under equal hypoxic load: 5s-60c caused persistent BrrA suppression during QS, suggesting chronic vagal blunting, whereas 10s-30c produced transient BrrD reductions, indicating acute reflex destabilization. These patterns indicate that high-frequency IH is associated with a persistent autonomic vulnerability phenotype, whereas longer hypoxic bouts transiently perturb reflex control. Few animal studies have compared matched IH structures [30]; our findings highlight hypoxia timing as a modulator of cardiovascular reflexes. Such alterations may be relevant to blood pressure instability observed in OSA and support baroreflex sensitivity as a temporally responsive marker of autonomic vulnerability [31, 32].

### Hematological adaptations and systemic implications

Peripheral blood markers differentiated systemic effects of IH structures. The 5s-60c group showed increased hematocrit, hemoglobin, and red cell distribution width, consistent with erythropoietic activation [33–36], whereas the 10s-30c group had reduced white blood cell and platelet counts, suggesting immunosuppression. These contrasting profiles reflect paradigm-dependent adaptation. Elevated hematocrit increases blood viscosity and vascular resistance, mechanisms associated with impaired nocturnal blood pressure regulation and autonomic imbalance [37, 38]. In this context, the non-dipping blood pressure pattern observed in the 5s–60c group, together with altered hematological profiles, represents a phenotype of heightened vascular–autonomic vulnerability. Although rarely used in OSA phenotyping, our findings suggest IH timing systematically shapes erythropoietic and immune responses. By isolating episode duration and frequency under equal hypoxic burden, our model provides mechanistic insight into how peripheral blood adaptations may interact with cardiovascular regulation in sleep-disordered breathing.

### Sleep fragmentation and cortical arousal

Both IH paradigms disrupted sleep but with distinct patterns. The 10s-30c group showed greater QS and PS fragmentation with PS rebound during recovery, reflecting heightened homeostatic pressure. By contrast, the 5s-60c group maintained more stable stages but displayed persistent PS beta activity and heightened arousability, suggesting latent hyperarousal without overt fragmentation. Clinically, this mirrors OSA phenotypes: some patients exhibit PS rebound after treatment, while others show increased cortical arousal despite preserved duration [39, 40]. Experimental and clinical evidence implicates central arousal circuits and cortical instability in IH-related disruption [11, 41, 42]. Increased beta activity is a well-established marker of cortical hyperarousal and heightened sympathetic tone [43, 44], and arousal-related sympathetic surges have been demonstrated in OSA during sleep disruption [45]. Chronic sympathetic activation, in turn, impairs endothelial function, promotes vascular stiffness, and contributes to blunted or non-dipping nocturnal blood pressure patterns [46, 47]. Together, these established pathways provide a mechanistic basis for our observation that high-frequency IH fosters subclinical hyperarousal and sustained sympathetic drive, representing a phenotype of impaired cardiovascular–autonomic regulation even in the absence of overt sleep fragmentation.

### Cognitive outcomes as correlates of blood pressure variability in intermittent hypoxia

OSA is linked to hypertension and cognitive decline, yet only about half of patients develop hypertension, and some maintain normal cognition [48, 49]. This heterogeneity suggests phenotype-specific mechanisms, including hypoxic load structure and sympathetic activation. In our study, the 10s-30c group lacked sustained hypertension but showed spatial memory deficits with reduced NeuN and elevated GFAP and NF-κB, consistent with neuronal loss and glial reactivity [50–53].

Clinical evidence indicates that cognitive decline in hypertension correlates more with blood pressure variability (BPV) than mean pressure [54]. Similarly, the 10s-30c group exhibited transient MAP elevations during IH that normalized afterward, suggesting higher BPV than the persistently elevated MAP of the 5s-60c group. Together, these findings indicate that neurocognitive impairment associated with longer hypoxic episode duration may be linked to blood pressure fluctuations rather than sustained hypertension. This interpretation aligns with clinical reports associating BPV with memory impairment and dementia and supports a conceptual framework in which cardiovascular and cognitive vulnerability in OSA may share overlapping, yet distinct, mechanistic pathways.

Prolonged hypoxic events are known to impose greater metabolic and inflammatory stress on neural tissue. Longer hypoxic events increase mitochondrial dysfunction and oxidative load, which activate NF-κB-mediated neuroinflammatory cascades and promote astrocytic reactivity [55–57]. Such mechanisms are consistent with the elevated GFAP and NF-κB and reduced NeuN observed in the 10s-30c paradigm and support the interpretation that extended hypoxic duration preferentially drives neural injury and cognitive impairment rather than sustained autonomic dysregulation.

### Translational perspective and future directions

Our findings underscore that the temporal structure of IH—beyond total hypoxic burden—critically shapes cardiovascular and autonomic outcomes. This concept parallels emerging clinical metrics such as hypoxic burden [58] and the cumulative time spent below an arterial oxygen saturation of 90% (T90) [59], which capture dimensions of nocturnal hypoxemia not reflected by event frequency alone. In conceptual terms, the 5s-60c paradigm reflects patterns characterized by frequent but relatively brief desaturations (higher AHI, lower per-event burden), whereas the 10s–30c group models fewer but more prolonged hypoxic events with greater desaturation severity per event. These distinct hypoxic patterns resemble clinically observed OSA phenotypes in which event frequency and desaturation duration differentially shape cardiovascular and autonomic vulnerability, independent of cumulative hypoxic exposure. By isolating episode duration and frequency under a fixed load, our model provides a mechanistic framework to aid interpretation of structure-aware hypoxemia metrics and to support future refinement of phenotyping approaches in sleep-disordered breathing. As summarized in the graphical abstract, short, frequent IH episodes primarily induced hypertension-related autonomic imbalance, whereas longer, fewer episodes were associated with neurocognitive alterations, highlighting distinct systemic vulnerability profiles that extend beyond conventional severity indices.

### Limitations

This study has several limitations. First, molecular and hematological measures were obtained only at Day 21, precluding evaluation of dynamic or early compensatory responses. Second, behavioral testing was restricted to spatial learning and exploration; broader cognitive and emotional domains warrant study. Third, although sample sizes detected major physiological differences, larger cohorts may resolve finer variability in molecular and hematological outcomes. Fourth, the IH paradigm—repetitive episodes during the light phase—does not fully replicate human OSA, particularly prolonged PS apneas. Finally, Peripheral capillary oxygen saturation (SpO₂) was not concurrently measured, limiting assessment of desaturation variability; future work combining oximetry with sleep-state tracking may better define how IH timing shapes systemic adaptation.

## Conclusion

The timing of hypoxic stress, in addition to its cumulative load, exerts distinct effects on downstream physiology. Our findings highlight that the temporal features of IH are important determinants of autonomic imbalance, blood pressure regulation, and neural vulnerability. Together, these results provide a mechanistic framework to help interpret the heterogeneity of cardiovascular and neurocognitive manifestations observed in sleep-disordered breathing.

## Supplementary information

Supplementary Information

## Data Availability

The data supporting the findings of this study were generated using a custom-designed physiological recording system developed in our laboratory. Due to technical constraints and identifiable information related to experimental procedures, the raw datasets cannot be made publicly available at this time. However, the authors are committed to transparency and will make the analyzed datasets available upon reasonable request. Full data sharing will be ensured upon acceptance of the manuscript.
