# Supplementary material for: Differential cardiovascular and autonomic responses to structurally distinct intermittent hypoxia paradigms in rats
Source: Hypertens Res. 2026 Mar 2;49(5):1659–72. doi: 10.1038/s41440-026-02588-7 (PMC13148984; doi:10.1038/s41440-026-02588-7)
Supplement: Supplementary file 1 — Supplementary Information [file 41440_2026_2588_MOESM1_ESM.docx]

**Supplementary Methods**

**Supplementary Methods**

**Animals and Housing Conditions**

Thirteen-week-old male Wistar-Kyoto rats (WKY; BioLASCO Taiwan Co., Ltd., Taipei, Taiwan; 300–330 g) were used in this study. Animals were housed in a sound-attenuated room under controlled conditions: 12:12-h light–dark cycle (lights on at 10:00; Zeitgeber time (ZT) 0 = lights on, ZT 12 = lights off), temperature 22 ± 2 °C, and relative humidity 40–70%. Standard chow and tap water were provided ad libitum. At 13 weeks of age, rats were implanted with telemeters for arterial blood pressure recordings. At 14 weeks of age, wireless electrodes were implanted for electroencephalography (EEG), electromyography (EMG), and electrocardiography (ECG). After surgery, rats received chlortetracycline and carprofen and were housed individually for at least 1 week of recovery. All procedures followed the National Institutes of Health Guide for the Care and Use of Laboratory Animals and were approved by the Institutional Animal Care and Use Committee of National Yang Ming Chiao Tung University (IACUC No. 1130408). Group sizes (n = 6–9) were determined from pilot data on mean arterial pressure during the quiet sleep phase, yielding a large effect size (Cohen’s f = 0.73). Post hoc power analysis (α = 0.05, one-way ANOVA, three groups) indicated >99% power to detect differences between intermittent hypoxia paradigms ^13^.

**Surgical implantation procedures**

Rats underwent sequential surgical procedures for the implantation of physiological recording devices. For arterial pressure measurement, animals were anesthetized with isopropanol, a midline incision was made to expose the abdominal aorta, and a small incision was created in the vessel wall to allow careful insertion of a catheter from a telemeter (HD-S10 implant, Data Sciences International, St. Paul, MN, USA). The catheter was secured with tissue adhesive, and the telemeter body was positioned in the abdominal cavity and sutured to adjacent muscle tissue for stability.

In a separate procedure, rats were anesthetized with pentobarbital (50 mg/kg, intraperitoneally) and placed in a stereotaxic apparatus. A scalp incision was made to expose the bregma and lambda landmarks, and six electrodes were implanted in the skull at the following coordinates for EEG recordings: frontal lobe (2.0 mm anterior to bregma, ±2.0 mm laterally), parietal lobe (2.0 mm posterior to bregma, ±2.0 mm laterally), and occipital lobe (6.0 mm posterior to bregma, ±2.0 mm laterally). An additional reference electrode was implanted 2 mm posterior to lambda. EMG signals were acquired via stainless-steel wires implanted bilaterally in the neck muscles, while ECG signals were acquired from wires implanted in the right forelimb and left waist muscles. All wires were connected to sensors transmitting data to the recording system^15-18^.

Following each procedure, rats received chlortetracycline and carprofen (5 mg/kg, subcutaneously) and were housed individually for a minimum recovery period of 1 week.

**Physiological signal recording methods**

**Custom wireless telemetry system for EEG, ECG, and EMG measurements**

Sampling rates were selected in accordance with those used in the literature. EEG, EMG, and ECG signals were sampled at 125, 250, and 500 Hz, respectively, amplified by factors of 1,000, 1,000, and 500, and band-pass filtered within the ranges of 0.16–48, 34–103, and 0.72–103 Hz, respectively. A sampling rate of 62.5 Hz was used for activity monitoring. Data were digitized and transmitted via 2.4-GHz high-frequency radio waves to the recording system (KY3, K&Y Lab, Taiwan)^15-18^.

**Telemeter for dynamic blood pressure recording**

A telemeter was used to continuously record dynamic blood pressure signals, transmitted in real time via radiofrequency waves to a receiving plate (CTR-86, Data Sciences International). Arterial pressure signals were band-limited to 0.06–2.4 Hz and sampled at 1024 Hz. Transmitted data were processed by a decoding unit (KY2, K&Y Lab) and synchronously forwarded to the recording system (KY3, K&Y Lab) ^15-18^.

**Data analysis methods**

**Frequency domain analysis and sleep phase classification**

EEG signals were analyzed using a 16-second window (1,024 points) with a 50% overlap (8-second shift), and EMG signals were processed using a nonoverlapping 2-second window (2,046 points). Baseline drift was removed via a nonparametric fast Fourier transform (FFT) approach, with a Hamming window applied to reduce spectral leakage. Power spectral density was calculated from 8-second segments (4,096 points) using FFT with corrections for sampling rate and windowing effects. EEG frequency bands were defined as alpha (10–13 Hz), beta (13–32 Hz), theta (4–12 Hz), and delta (0.5–4 Hz) ^15-18^.

Sleep status for each rat was determined for each 1-hour window. Classification as awake, quiet sleep (QS), or paradoxical sleep (PS) was based on mean EEG power frequency and EMG power values. Windows with both parameters above thresholds were scored as wakefulness; both below thresholds were scored as QS; mean EEG power frequency above threshold with EMG power below threshold was scored as PS. Thresholds were set by an experienced researcher based on behavioral observations ^15-18^.

**ECG and blood pressure signal processing**

For heart-rate variability analysis, ECG signals were processed using a QRS detection algorithm to identify complexes, and a template was created based on mean amplitude and duration. Complexes exceeding three times this standard were considered noise or ectopic beats. R-peaks were identified, RR intervals calculated, and intervals exceeding six times the standard were excluded.

RR intervals and arterial pressure data were resampled at 64 Hz, baseline drift removed using FFT with a Hamming window, and power spectral density calculated from 8-second segments (4,096 points). Spectral power was quantified as total power, low-frequency (LF, 0.06–0.6 Hz), high-frequency (HF, 0.6–2.4 Hz), LF%, and the low-frequency band of blood pressure variability (BLF, 0.06–0.6 Hz) ^15-18^.

Based on Task Force standards and prior laboratory studies, HF was interpreted as an index of cardiac vagal activity, LF% as cardiac sympathetic activity and BLF as vascular sympathetic activity. For Baroreflex sensitivity (BRS) analysis, beat-to-beat MAP and RR interval time series were aligned. Sequences of ≥3 consecutive beats with concordant increases or decreases in MAP and RR intervals were selected. Regression lines were fitted to each sequence; sequences with r > 0.85 were accepted. The mean slope of ascending MAP sequences was defined as BrrA (baroreflex sensitivity during rising pressure), while the mean slope of descending MAP sequences was defined as BrrD (baroreflex sensitivity during falling pressure). ^15-18^.

**The IH model**

The IH model used in this experiment was adapted from that described by Fletcher et al. [20] to simulate various patterns of IH associated with obstructive sleep apnea. During the sleep phase (light period), rats were placed in an acrylic chamber (30 cm long, 12 cm wide, 12 cm high; volume 4.3 L) for 8 hours. Within the chamber, 100% nitrogen gas was injected for approximately 30 seconds to reduce the oxygen concentration to 4–6%. A hypoxic state was maintained for either 5 seconds or 10 seconds, depending on group assignment, after which the chamber was infused with air to restore the oxygen level to 21%. Oxygen cycling was automatically controlled using a micro:bit-based system (micro:bit V2.2, Micro:bit Educational Foundation). The micro:bit opened an electric valve to inject nitrogen into the chamber, while oxygen concentration was continuously monitored by an oxygen sensor (Grove–Gas Sensor [O₂], Seeed Studio). Upon reaching the target hypoxic concentration, the nitrogen valve was closed. After the designated hypoxia duration, the micro:bit opened an air valve to restore oxygen concentration to 21%, and the cycle repeated. The cycle length was 2 minutes in the 10s–30c group and 1 minute in the 5s–60c group. This protocol was applied daily for 21 consecutive days ^19,20^.

**Behavioral test**

**Eight-arm maze test**

The eight-arm maze test was used to evaluate spatial learning ability. The test apparatus was placed directly on the floor, and a small water-filled container (3 cm diameter, 1 cm depth) was positioned at the end of each arm. Prior to the experiment, rats were water-deprived for 24 hours to enhance motivation. Training was conducted over 3 consecutive days, with one session per day. In each training session, the rat was placed in the center of the maze and allowed to explore and search for water for 10 minutes.

On the fourth day, a formal test was conducted. The test ended when the rat had visited all eight arms and located all water sources or when the 10-minute time limit had been reached. Reentries into previously visited arms were counted as errors. The number of correct arms entered before the first error was recorded as the correct score ^21^. Additional parameters included: (1) total exploration time (general activity and motivation), (2) number of arms visited (exploratory behavior), (3) number of correct entries (successful spatial memory retrieval), (4) number of errors (working memory deficits), (5) correct rate (correct entries / total entries), and (6) error rate (error entries / total entries) as integrated indices of spatial learning ability.

**Collection of blood samples**

Before sacrifice, blood samples were collected from the carotid artery under isoflurane anesthesia. Under sterile conditions, a midline neck incision was made to expose the carotid artery, which was then cannulated with an appropriate gauge needle to collect blood directly into a microcentrifuge tube. Samples were allowed to settle for 30 minutes at room temperature and subsequently centrifuged at 3,000 rpm and 4 °C for 10 minutes. The supernatant was stored at −80 °C until further analysis.

Whole-blood samples were analyzed on-site at the Laboratory Animal Center, National Yang Ming Chiao Tung University, Taiwan, using an automated hematology analyzer (Sysmex XT-1800iv) to measure red blood cell count, white blood cell count, platelet count, and related hematological parameters. Serum samples were sent to Union Clinical Laboratory (Taiwan) for biochemical analysis of aspartate aminotransferase, alanine aminotransferase, blood urea nitrogen, and creatinine concentrations.

**Western blotting**

Magnetic beads were used to homogenize tissue samples in radioimmunoprecipitation assay buffer supplemented with protease and phosphatase inhibitors (ratio 1:10:0.1:0.1, tissue:radioimmunoprecipitation assay buffer:protease inhibitor:phosphatase inhibitor, μL). Homogenates were centrifuged at 12,000 × g for 15 minutes at 4 °C. Equal volumes (20 μL) of homogenate were loaded per lane for SDS–PAGE, and GAPDH was used as the internal loading control for normalization.

Proteins were transferred onto either polyvinylidene difluoride or nitrocellulose membranes, depending on the target protein. Membranes were blocked for 1 hour at room temperature using either 5% nonfat milk or 5% bovine serum albumin in 0.05% phosphate-buffered saline containing Tween-20, depending on the antibody’s background sensitivity. Primary antibodies were incubated overnight at 4 °C and included anti-NF-κB (1:1,000, A19653), anti-GPX4 (1:1,000, AB125066), anti-GFAP (1:1,000, MAB360), anti-COX-2 (1:1,000, A1253), anti-ionized calcium-binding adapter molecule 1 (IBA-1, 1:1,000, A12391), anti-NeuN (1:1,000, A0951), anti-BDNF (monoclonal, 1:1,000, ab108319), anti-BDNF (polyclonal, 1:1,000, ab226843), and anti-glyceraldehyde 3-phosphate dehydrogenase (GAPDH, 1:60,000, AC033).

Following washes with phosphate-buffered saline with Tween-20, membranes were incubated with horseradish peroxidase–conjugated secondary antibodies (1:5,000, RA-BZ202) for 80 minutes at room temperature. Signals were detected using enhanced chemiluminescence and imaged. Band intensities were quantified using Multi Gauge software (Fujifilm), normalized to GAPDH, and expressed as relative expression values for each group.

The monoclonal BDNF antibody (ab108319) predominantly recognized a band at approximately 37–38 kDa, corresponding to precursor BDNF (proBDNF). The polyclonal BDNF antibody (ab226843) revealed multiple bands, one of which (approximately 28 kDa) was consistently detected in brain tissue and selected for interpretation. The mature BDNF band (approximately 14–17 kDa) was not used because of weak or inconsistent detection. Differences in observed molecular weights are attributable to distinct epitope recognition and the presence of different BDNF isoforms and processing stages.

**Supplementary Figure**


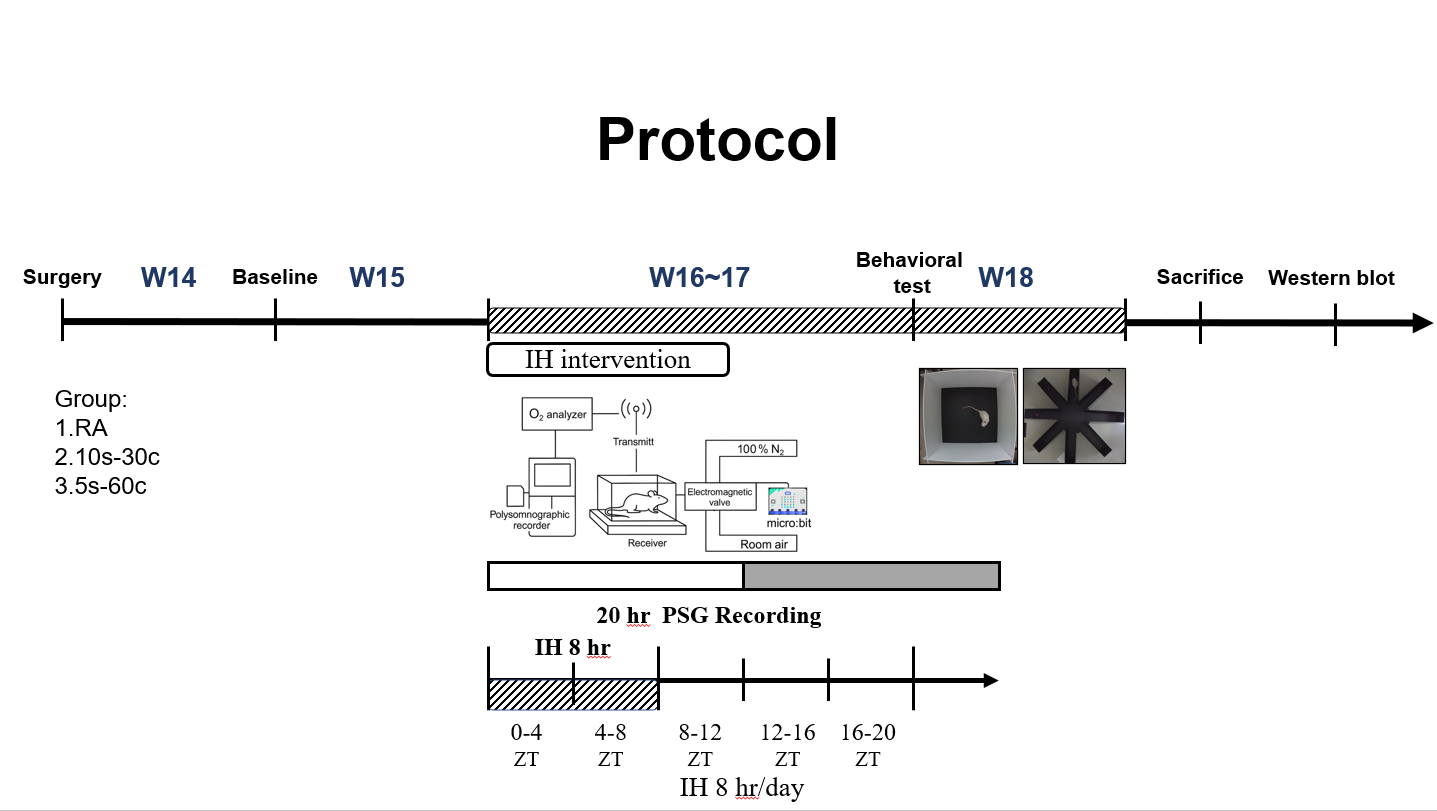


**Supplementary Figure S1. Experimental protocol.**

A total of 37 male Wistar Kyoto rats were obtained at 13 weeks of age. Arterial pressure telemetry devices and sleep monitoring devices were implanted at 13 and 14 weeks, respectively, with one week of recovery after each surgery. Baseline physiological signals were recorded at 15 weeks of age. At 16 weeks, rats underwent a 3-week intermittent hypoxia (IH) intervention. Animals were randomly assigned to one of three groups: 10s–30c group (10-second hypoxic episodes at 30 cycles/hour for 8 h/day), 5s–60c group (5-second episodes at 60 cycles/hour for 8 h/day), and a control group exposed to room air under identical chamber conditions. Physiological recordings were performed during the second week of the intervention. In the third week, behavioral assessments—including the open field test and the eight-arm maze—were conducted. At the completion of the protocol, rats were sacrificed, and blood and brain tissues were harvested for biochemical analyses.


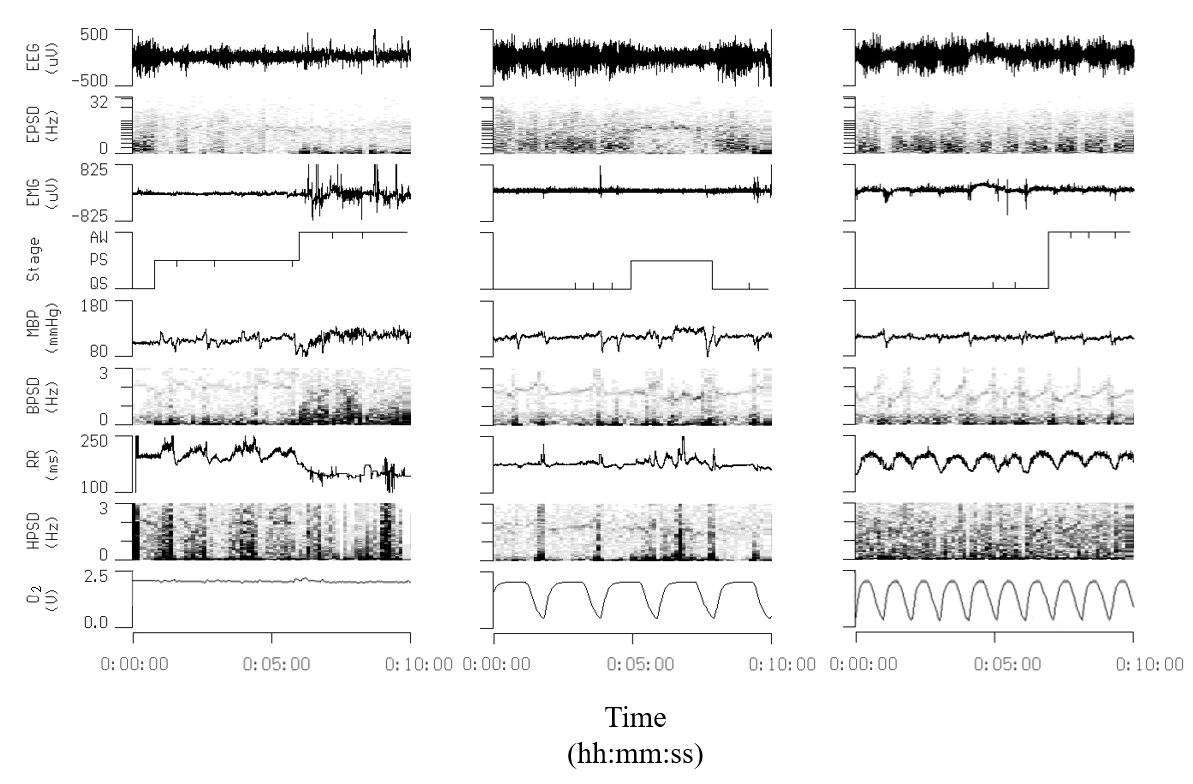


**Supplementary Figure S2. Representative raw physiological signals under IH.**

Ten-minute recordings during the light phase from each group are shown. From top to bottom: EEG with event-related power spectral density (EPSD), EMG, sleep stage (AW: awake; QS: quiet sleep; PS: paradoxical sleep), mean blood pressure (MBP) with blood pressure spectral density (BPSD), RR interval with power spectral density of heart rate (HPSD)”, and ambient oxygen concentration.


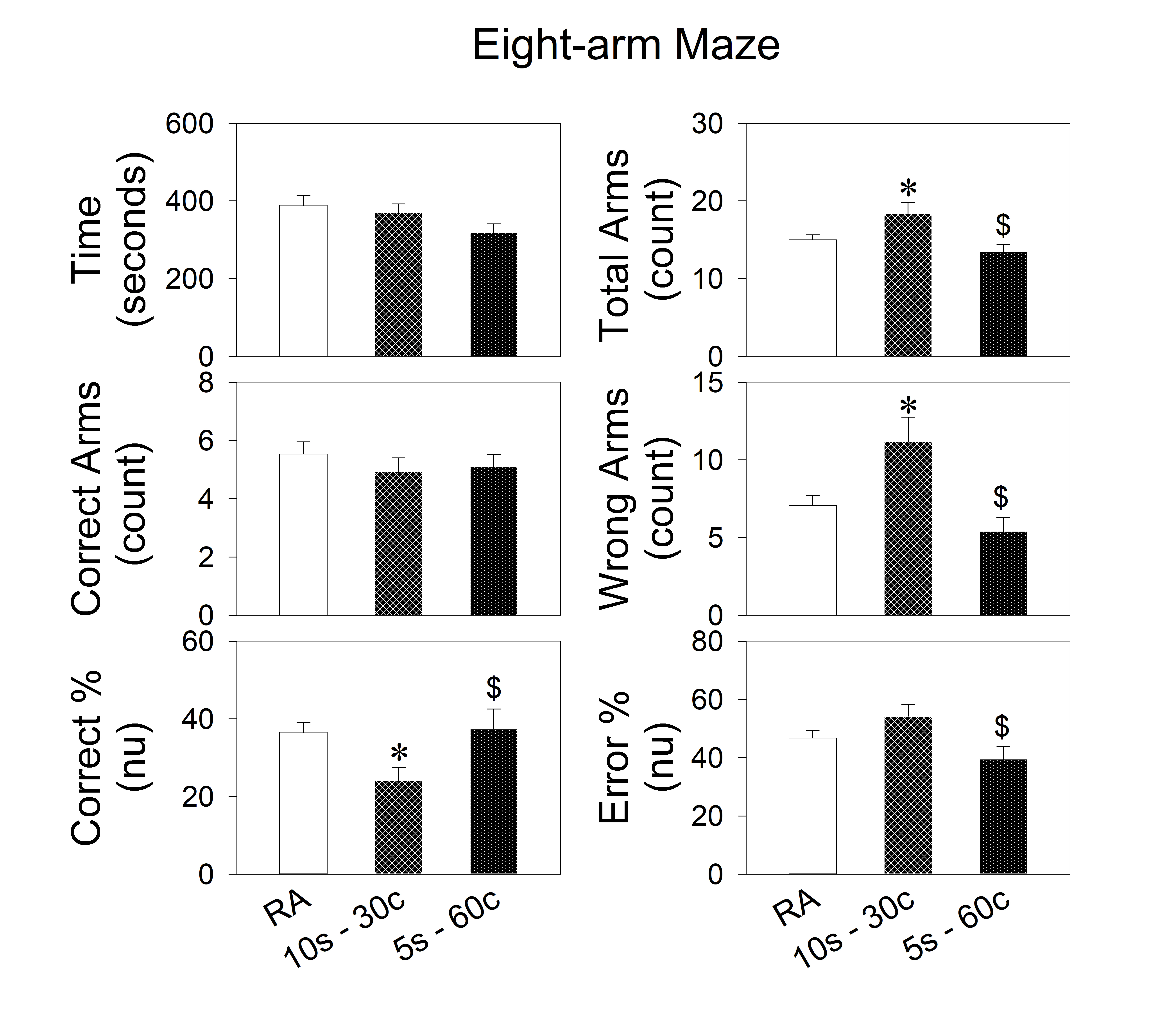


Supplementary Figure S3. Effects of IH on spatial memory in eight-arm maze test.

Effects of intermittent hypoxia (IH) on spatial memory performance in the eight-arm maze test. Three groups were compared: control (room air, RA), 10s–30c (10-second hypoxia episodes at 30 cycles/hour), and 5s–60c (5-second hypoxia episodes at 60 cycles/hour). The measured parameters were the time required to complete the test, total number of arm entries, number of arms entered before the first repetition, number of repeated arm entries, ratio of correct arm entries to total entries, and ratio of incorrect arm entries to total entries. Data are expressed as mean ± standard error of the mean. Sample sizes: control (n = 15), 10s–30c (n = 12), and 5s–60c (n = 10). *p < 0.05 vs. control; ^$^p < 0.05 vs. 10s–30c (one-way ANOVA with least-squares difference [LSD] post hoc test).


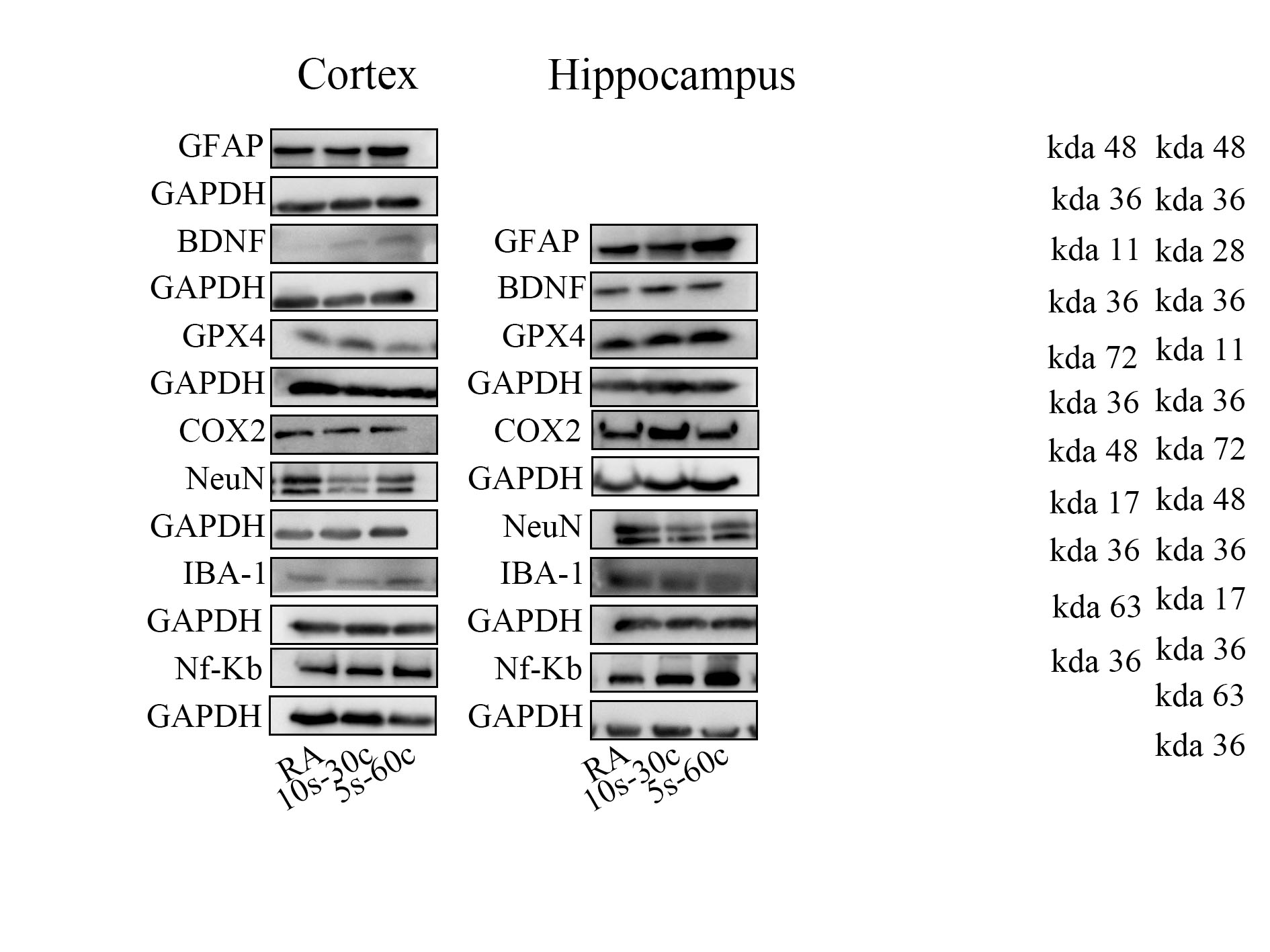

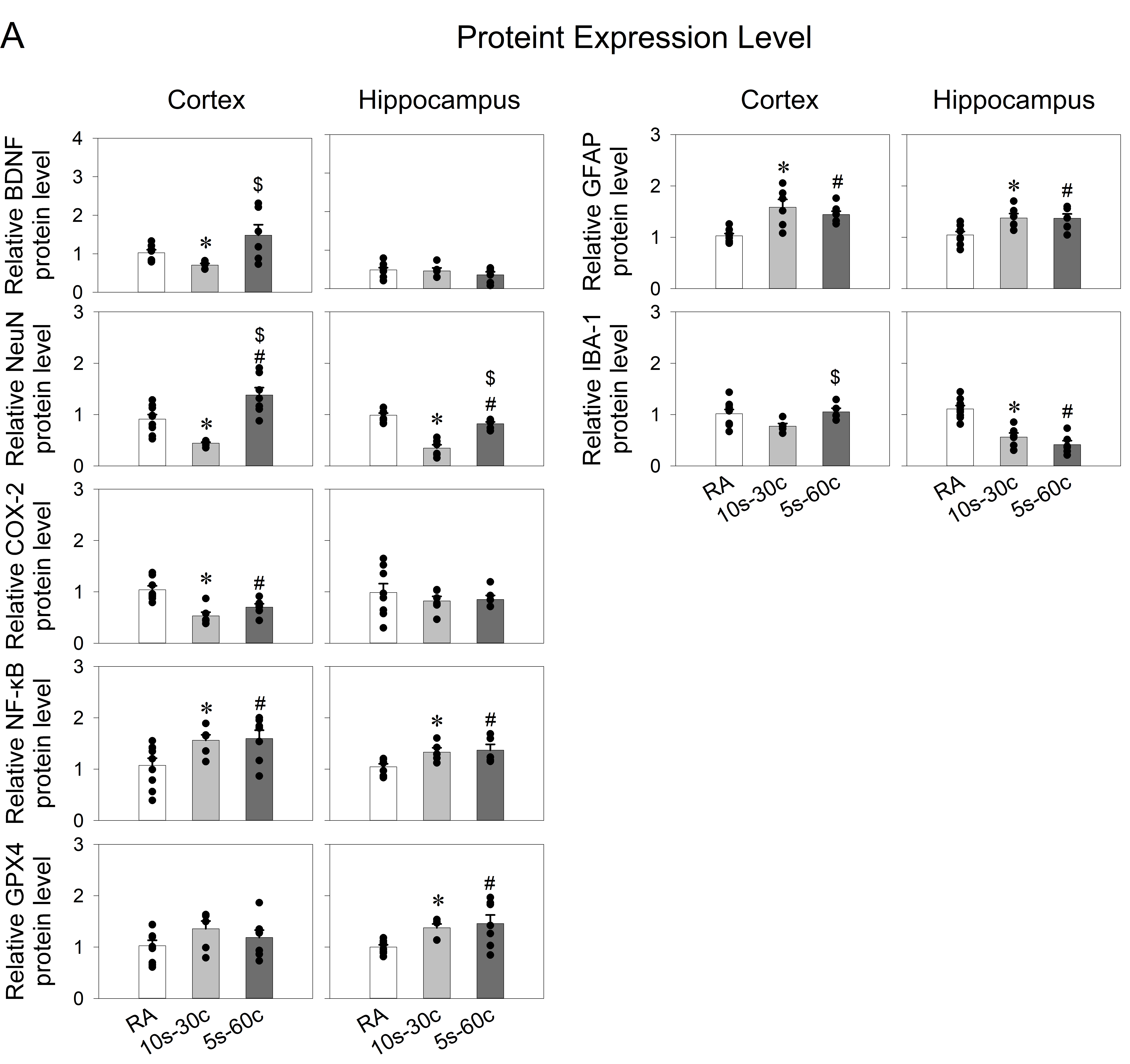


B

**Supplementary Figure S4.** **Concentrations of inflammatory, oxidative stress, and microglial activation markers in cortex and hippocampus after 3 weeks of IH exposure in control, 10s–30c, and 5s–60c groups.**

(A) Relative concentrations of brain-derived neurotrophic factor (BDNF), neuronal nuclei (NeuN), glial fibrillary acidic protein (GFAP), nuclear factor kappa-light-chain-enhancer of activated B cells (NF-κB), cyclooxygenase-2 (COX-2), glutathione peroxidase 4 (GPX4), and ionized calcium-binding adapter molecule 1 (IBA-1) in the cortex and hippocampus after 21 days of intermittent hypoxia (IH) exposure. (B) Representative Western blot images for the corresponding markers in the cortex and hippocampus. Concentrations were normalized to glyceraldehyde-3-phosphate dehydrogenase (GAPDH) as an internal control. Groups: control (room air); 10s–30c (10-second hypoxia episodes at 30 cycles/hour); and 5s–60c (5-second hypoxia episodes at 60 cycles/hour). IH exposure was administered for 8 hours per day over 21 consecutive days. Data are presented as mean ± standard error of the mean. Sample sizes: control (n = 9), 10s–30c (n = 6), and 5s–60c (n = 7). Statistical significance: *p < 0.05 for 10s–30c vs. control; ^#^p < 0.05 for 5s-60c vs. control; ^$^p < 0.05 for 5s-60c vs. 10s-30c (one-way ANOVA with Fisher’s LSD post hoc test).

| Supplementary Table 1. Mean ± SEM at ZT 0–4 and ZT 16–20, and corresponding differences in cardiovascular and autonomic parameters | | | | | | | | | | | |  |
| --- | --- | --- | --- | --- | --- | --- | --- | --- | --- | --- | --- | --- |
|  | RA | | |  | 10s-30c | | |  | 5s-60c | | |  |
|  | ZT 0-4 | ZT16-20 | differences |  | ZT 0-4 | ZT16-20 | differences |  | ZT 0-4 | ZT16-20 | differences |  |
| AW-MAP | 116.50 ± 3.10 | 126.81 ± 1.71 | 10.31 ± 3.54 |  | 123.24 ± 3.98 | 125.27 ± 3.89 | 2.03 ± 5.57 |  | 118.94 ± 2.74 | 126.46 ± 1.91 | 7.51 ± 3.34 |  |
| AW-BLF | 2.70 ± 0.17 | 2.02 ± 0.17 | -0.68 ± 0.24 |  | 2.38 ± 0.09 | 1.86 ± 0.08 | -0.52 ± 0.12 |  | 2.54 ± 0.09 | 2.05 ± 0.05 | -0.48 ± 0.10 |  |
| AW-RR | 177.63 ± 3.69 | 167.27 ± 5.87 | -10.36 ± 6.93 |  | 170.30 ± 3.53 | 156.69 ± 2.74 | -13.60 ± 4.47 |  | 165.38 ± 3.68 | 156.29 ± 1.31 | -9.09 ± 3.90 |  |
| AW-HF | 1.50 ± 0.20 | 0.96 ± 0.20 | -0.54 ± 0.28 |  | 1.48 ± 0.10 | 0.43 ± 0.13 | -1.05 ± 0.17 |  | 1.23 ± 0.24 | 0.25 ± 0.15 | -0.98 ± 0.28 |  |
| AW-LF% | 85.89 ± 1.97 | 80.49 ± 2.43 | -5.40 ± 3.13 |  | 86.79 ± 0.86 | 83.98 ± 0.87 | -2.81 ± 1.22 |  | 85.90 ± 1.45 | 82.53 ± 0.98 | -3.36 ± 1.75 |  |
|  |  |  |  |  |  |  |  |  |  |  |  |  |
| QS-MAP | 109.98 ± 2.04 | 112.62 ± 1.27 | 2.64 ± 2.40 |  | 117.45 ± 3.08 | 109.27 ± 2.20 | -8.18 ± 3.78 |  | 118.63 ± 1.66 | 117.16 ± 0.63 | -1.47 ± 1.78 |  |
| QS-BLF | 0.65 ± 0.05 | 0.50 ± 0.09 | -0.15 ± 0.11 |  | 0.97 ± 0.09 | 0.32 ± 0.09 | -0.64 ± 0.12 |  | 1.03 ± 0.09 | 0.70 ± 0.09 | -0.33 ± 0.12 |  |
| QS-RR | 204.53 ± 3.85 | 198.90 ± 3.11 | -5.63 ± 4.95 |  | 187.71 ± 2.99 | 192.54 ± 4.06 | 4.84 ± 5.04 |  | 172.53 ± 3.42 | 186.07 ± 1.87 | 13.54 ± 3.90 |  |
| QS-HF | 1.44 ± 0.16 | 1.32 ± 0.18 | -0.12 ± 0.24 |  | 1.64 ± 0.15 | 1.14 ± 0.14 | -0.50 ± 0.21 |  | 1.42 ± 0.10 | 1.01 ± 0.16 | -0.41 ± 0.18 |  |
| QS-LF% | 56.00 ± 2.42 | 60.69 ± 2.57 | 4.69 ± 3.54 |  | 70.55 ± 1.63 | 56.81 ± 1.35 | -13.75 ± 2.11 |  | 73.43 ± 1.35 | 65.33 ± 1.05 | -8.09 ± 1.71 |  |
|  |  |  |  |  |  |  |  |  |  |  |  |  |
| PS-MAP | 119.73 ± 2.04 | 116.32 ± 1.53 | -3.41 ± 2.55 |  | 123.99 ± 4.18 | 113.89 ± 2.65 | -10.10 ± 4.95 |  | 122.74 ± 3.44 | 120.34 ± 1.94 | -2.40 ± 3.95 |  |
| PS-BLF | 1.62 ± 0.07 | 1.25 ± 0.17 | -0.37 ± 0.18 |  | 1.76 ± 0.11 | 0.87 ± 0.04 | -0.89 ± 0.12 |  | 2.06 ± 0.12 | 1.04 ± 0.05 | -1.01 ± 0.12 |  |
| PS-RR | 215.58 ± 2.99 | 203.74 ± 4.33 | -11.85 ± 5.26 |  | 197.45 ± 4.23 | 197.74 ± 3.84 | 0.29 ± 5.71 |  | 178.05 ± 3.33 | 196.78 ± 1.80 | 18.74 ± 3.78 |  |
| PS-HF | 2.25 ± 0.20 | 1.78 ± 0.20 | -0.47 ± 0.29 |  | 2.19 ± 0.12 | 1.15 ± 0.14 | -1.03 ± 0.18 |  | 1.80 ± 0.09 | 1.52 ± 0.18 | -0.28 ± 0.21 |  |
| PS-LF% | 70.28 ± 2.06 | 71.52 ± 2.47 | 1.24 ± 3.22 |  | 79.97 ± 1.38 | 71.88 ± 2.29 | -8.09 ± 2.67 |  | 81.68 ± 0.92 | 71.15 ± 1.08 | -10.53 ± 1.42 |  |
| Table 1. Mean ± SEM at ZT 0–4 and ZT 16–20, and corresponding differences in cardiovascular and autonomic parameters Data represent physiological signals recorded during the second week of a two-week intermittent hypoxia (IH) protocol. Values are expressed as mean ± SEM and are shown for two 4-h Zeitgeber time windows: ZT 0–4, corresponding to the early phase during IH exposure, and ZT 16–20, corresponding to the post-IH recovery period when hypoxic cycling was no longer applied. The differences column denotes the arithmetic difference between ZT 16–20 and ZT 0–4 within the same experimental group. Parameters include arterial pressure and autonomic indices measured during active wakefulness (AW), quiet sleep (QS), and paradoxical sleep (PS). RA, room air; MAP, mean arterial pressure; BLF, low-frequency component of blood pressure variability; RR, RR interval; HF, high-frequency component of heart rate variability; LF%, normalized low-frequency component of heart rate variability. | | | | | | | | | | | |  |
|  |  |  |  |  |  |  |  |  |  |  |  |  |
|  |  |  |  |  |  |  |  |  |  |  |  |  |
|  |  |  |  |  |  |  |  |  |  |  |  |  |
|  |  |  |  |  |  |  |  |  |  |  |  |  |
|  |  |  |  |  |  |  |  |  |  |  |  |  |
|  |  |  |  |  |  |  |  |  |  |  |  |  |

| Supplementary Table 2  Comparison of Hematological Parameters among Different Intermittent Hypoxia (IH) Interventions | | | | | | | | | | |
| --- | --- | --- | --- | --- | --- | --- | --- | --- | --- | --- |
|  |  |  | RA | |  | 10s-30c | |  | 5s-60c | |
|  |  |  | mean | SE |  | mean | SE |  | mean | SE |
| RBC | 106/uL |  | 7.05 | 0.53 |  | 6.67 | 0.38 |  | 7.45 | 0.30 |
| HGB | g/dL |  | 11.93 | 0.79 |  | 10.96 | 0.61 |  | 13.65 ^$^ | 0.36 |
| HCT | % |  | 34.05 | 2.06 |  | 30.82 | 1.29 |  | 39.32 ^*^ ^$^ | 1.93 |
| MCV | fL |  | 44.69 | 1.03 |  | 44.71 | 0.74 |  | 46.25 | 1.08 |
| MCH | pg |  | 15.70 | 0.17 |  | 15.82 | 0.08 |  | 15.78 | 0.25 |
| MCHC | g/dL |  | 34.23 | 0.73 |  | 34.64 | 0.43 |  | 32.32 | 0.96 |
| WBC | 103/uL |  | 4.13 | 0.51 |  | 2.49 ^*^ | 0.18 |  | 4.2 ^$^ | 0.60 |
| PLT | 103/uL |  | 489.50 | 28.43 |  | 379.18 ^*^ | 32.06 |  | 481.13 ^$^ | 37.49 |
| RDW-CV | % |  | 18.50 | 0.37 |  | 18.80 | 0.54 |  | 20.03 ^*^ | 0.33 |
| NEUT% | % |  | 41.93 | 2.07 |  | 41.20 | 3.37 |  | 43.25 | 1.72 |
| LYMPH% | % |  | 50.58 | 2.08 |  | 49.27 | 3.48 |  | 47.44 | 1.82 |
| MONO% | % |  | 3.81 | 0.32 |  | 4.29 | 0.37 |  | 4.12 | 0.46 |
| EO% | % |  | 0.76 | 0.07 |  | 0.87 | 0.12 |  | 0.67 | 0.08 |
| BASO% | % |  | 0.06 | 0.03 |  | 0.02 | 0.02 |  | 0.03 | 0.02 |
| Data are expressed as mean ± SE. RA (n = 11), control group exposed to room air (normoxia); 10s–30c (n = 13), intermittent hypoxia (IH) with 10-second hypoxia episodes delivered at 30 cycles per hour; 5s–60c (n = 11), IH with 5-second hypoxia episodes delivered at 60 cycles per hour. Both IH protocols were applied 8 h/day for 21 consecutive days. Abbreviations used in the table: RBC (red blood cell count), HGB (hemoglobin concentration), HCT (hematocrit), MCV (mean corpuscular volume), MCH (mean corpuscular hemoglobin), MCHC (mean corpuscular hemoglobin concentration), WBC (white blood cell count), PLT (platelet count), RDW-CV (red cell distribution width–coefficient of variation), NEUT% (neutrophil percentage), LYMPH% (lymphocyte percentage), MONO% (monocyte percentage), EO% (eosinophil percentage), BASO% (basophil percentage). Statistical significance: *p < 0.05 vs. RA; ^$^p < 0.05 vs. 10s–30c. One-way ANOVA followed by LSD post hoc test was used for group comparisons. | | | | | | | | | | |
|  |  |  |  |  |  |  |  |  |  |  |
|  |  |  |  |  |  |  |  |  |  |  |
|  |  |  |  |  |  |  |  |  |  |  |
|  |  |  |  |  |  |  |  |  |  |  |
|  |  |  |  |  |  |  |  |  |  |  |
